# Diagnostic delay, misdiagnosis, and patient-reported psychosocial burden among Chinese individuals with McCune–Albright syndrome: a national cross-sectional survey

**DOI:** 10.1186/s13023-026-04507-y

**Published:** 2026-07-15

**Authors:** Ke Yuan, Yilin Zhu, Yonghua Chen, Jianfang Zhu, Yanlan Fang, Chunlin Wang

**Affiliations:** Department of Pediatrics, The First Affiliated Hospital, Zhejiang University School of Medicine, No. 79 Qingchun Road, Hangzhou, 310003 Zhejiang China

**Keywords:** McCune–Albright syndrome, Rare disease, Diagnostic delay, Fibrous dysplasia, Psychosocial impact, China

## Abstract

**Background:**

McCune–Albright Syndrome (MAS) is a rare, mosaic genetic disorder characterized by fibrous dysplasia of bone, endocrine hyperfunction, and skin pigmentation. Despite increasing clinical recognition, real-world data on diagnostic timelines, disease burden, and psychosocial impact among MAS patients—particularly in China—remain scarce.

**Methods:**

We conducted a national, cross-sectional survey of Chinese MAS patients between November 2024 to June 2025 using a structured 95-item online questionnaire disseminated via the McCune–Albright Syndrome Clinical Research Consortium (MASCRC) database and the Bone Care Center patient community. Responses were de-duplicated and managed within the MASCRC data platform. Data were collected on demographics, initial symptoms, diagnostic delay, treatment history, quality of life, psychological distress, and support needs. Descriptive statistics, chi-square tests, and Mann–Whitney U tests were performed.

**Results:**

A total of 152 valid responses were analyzed from MAS patients across 28 of 34 provincial-level regions, including 94 females (61.84%) and 58 males (38.16%), with a median age of 10.10 years (IQR: 7.80–15.00). The most common first symptoms were bone involvement (61.18%), peripheral precocious puberty (36.84%), and café-au-lait pigmentation (37.50%). Initial hyperthyroidism and Cushing syndrome were reported in 3.95% and 1.32% of cases, respectively. Median age at symptom onset was 5.50 years; median age at diagnosis was 6.00 years. Although the majority of patients were diagnosed within 1 year (median delay: 0 months, IQR: 0–6), 21.05% experienced a diagnostic delay of ≥ 1 year, and 27.63% reported prior misdiagnosis. *GNAS* mutation testing was performed in 48.68% of patients, with a detection rate of 36.49%. Long-term consistent treatment was reported by 37.00% of respondents, with regular follow-up (62.50%), medication (38.82%), and surgery (36.18%) being the most common strategies. Impact on daily life was substantial: 46.05% of patients reported moderate to severe disruption in education or employment, and family/economic burden was rated most severe (mean score 3.21/5). Self-reported psychological distress was common, with 78.29% reporting anxiety-related symptoms and 37.50% reporting depressive symptoms, yet only 15.79% accessed professional support. Over 90% expressed unmet needs for expert medical resources, while 79.61% desired improved insurance reimbursement, and 46.05% wished for peer-support platforms.

**Conclusions:**

MAS patients in China report substantial clinical and psychosocial burdens, with a subset experiencing prolonged diagnostic journeys and misdiagnoses. These findings highlight a need for earlier recognition, integrated multidisciplinary care, and structured patient support.

**Supplementary Information:**

The online version contains supplementary material available at https://doi.org/10.1186/s13023-026-04507-y.

## Introduction

McCune–Albright syndrome (MAS) is a rare mosaic genetic disorder caused by postzygotic activating variants in the *GNAS* gene (NM_000516), resulting in constitutive activation of Gsα signaling and excess cyclic AMP production. The syndrome is classically characterized by fibrous dysplasia (FD) of bone, café-au-lait skin pigmentation, and autonomous endocrine hyperfunction, most commonly peripheral precocious puberty (PPP). In China, diagnosis of MAS currently relies primarily on internationally accepted clinical criteria, supported by molecular confirmation when available. However, the clinical presentation is highly heterogeneous, ranging from isolated skeletal lesions to severe multisystem disease involving endocrine, dermatologic, and skeletal complications. [1–2].

The rarity and phenotypic variability of MAS frequently result in delayed diagnosis and fragmented care [3]. Previous studies from Europe and Asia have reported substantial diagnostic delays, often ranging from several months to multiple years, with many patients initially receiving alternative diagnoses before MAS is recognized. In addition to diagnostic challenges, patients commonly experience chronic pain, skeletal deformities, endocrine dysfunction, repeated medical interventions, and uncertainty regarding disease progression.

In recent years, increasing attention has been paid to the psychosocial consequences of MAS. Studies from FD/MAS cohorts have demonstrated impaired quality of life, reduced physical functioning, anxiety-related symptoms, negative illness perceptions, and social participation difficulties. Nevertheless, most published studies originate from Europe or North America, and patient-reported data from Asia remain limited [4–6].

In China, systematic investigations into the clinical presentation, diagnostic journey, and psychosocial needs of MAS patients are lacking. Most existing literature focuses on small retrospective analyses, providing limited insight into the broader patient experience [4–5]. Understanding these patients’ real-world challenges, clinical burdens, and unmet support needs is essential for improving MAS care and informing healthcare policies.

Therefore, we conducted a national cross-sectional study aiming to comprehensively characterize the clinical features, diagnostic delays, psychosocial burdens, and patient-reported unmet needs among MAS patients via the McCune–Albright Syndrome Clinical Research Consortium (MASCRC) database and the Bone Care Center patient community. This study seeks to inform targeted interventions and multidisciplinary care strategies tailored specifically to the MAS patient community.

## Methods

### Study design and participants

We conducted a nationwide, cross-sectional, survey-based study in China from November 2024 to June 2025 to systematically characterize the clinical characteristics, diagnostic delays, psychosocial impacts, and unmet medical needs of patients with MAS. The survey was disseminated through MASCRC data from the China MAS Diagnosis and Treatment Collaborative Innovation Alliance and Bone Care Center, a nationwide MAS patient support community.

Participants were eligible if they:

1. Had a confirmed diagnosis of MAS.
2. Were capable of completing the online questionnaire independently or with guardian assistance.
3. When available, patients completed the survey themselves; otherwise, a primary caregiver completed the questionnaire on the patient’s behalf.

A total of 152 valid responses were collected from patients across 28 of 34 provincial-level administrative regions in China.

Survey Instrument

We developed a comprehensive, structured questionnaire to capture domains that are not fully covered by existing generic patient-reported outcome measures (PROMs), including the diagnostic pathway (e.g., misdiagnosis and time to diagnosis), patterns of care access, and disease-specific support needs in China. Item generation was informed by an extensive literature review, expert consultation (pediatric endocrinology, orthopedics, and rare disease management), and prior international MAS patient experience work. The questionnaire comprised 95 items organized into five main domains:

1. Demographic and socioeconomic characteristics: Age, gender, education, employment status, family income, and geographic location.
2. Clinical presentation and medical history: Age at symptom onset and diagnosis, types of initial and subsequent symptoms (e.g., precocious puberty, FD), diagnostic processes including misdiagnosis experiences, genetic testing results (*GNAS* mutation status), and ongoing treatment modalities.
3. Impact on daily life: Degree of disruption in education, employment, mobility, self-care, family finances, and overall quality of life, assessed using standardized five-point Likert scales.
4. Psychological well-being: Self-reported psychological distress, including anxiety, depressive symptoms, low self-esteem, and social withdrawal, along with prior access to psychological support.
5. Patient needs and support preferences: Assessment of unmet medical care needs, preferred formats of patient education (e.g., short videos, live lectures), desire for professional psychological services, and interest in peer-support communities.

The draft questionnaire was pilot-tested with 10 patients/caregivers to assess comprehensibility and face validity, and refined accordingly. However, formal psychometric validation (e.g., reliability, construct validity, test–retest) was not performed and is planned in future work, alongside cross-cultural adaptation of validated PROMs (e.g., PedsQL, HADS/PROMIS) for use in Chinese MAS populations.

### Diagnostic criteria

MAS was defined according to current international consensus criteria, which are also adopted in current clinical practice in China as the presence of fibrous dysplasia together with at least one characteristic extra-skeletal manifestation (e.g., peripheral precocious puberty, café-au-lait skin pigmentation, hyperthyroidism, growth hormone excess, phosphate wasting, or hypercortisolism), or a documented diagnosis established by a specialist center.

Although the survey was distributed through a broader MAS patient community, only respondents meeting MAS diagnostic criteria were included in the final analysis. Individuals with isolated fibrous dysplasia without evidence of MAS were excluded.

### Recruitment and registry

Participants were recruited through the McCune–Albright Syndrome Clinical Research Consortium (MASCRC) database and the Bone Care Center patient community, a nationwide patient-support organization. Patients entered these platforms through physician referral, patient self-registration, or family registration after receiving a clinical diagnosis of MAS.

Participation in the survey was voluntary and anonymous. Electronic informed consent was obtained before questionnaire initiation. For participants younger than 18 years, consent was obtained from parents or legal guardians.

### Questionnaire development

A structured 95-item questionnaire was developed specifically for this study through a multi-step process. First, a literature review was conducted to identify domains relevant to diagnostic experiences, quality of life, psychosocial well-being, and healthcare needs among patients with rare diseases and MAS. Second, the preliminary questionnaire was reviewed by pediatric endocrinologists, rare disease specialists, and patient representatives. Third, pilot testing was performed among 10 MAS patients and caregivers to assess clarity, readability, and face validity.

The final questionnaire included five predefined domains: (1) demographic and socioeconomic characteristics; (2) clinical manifestations and diagnostic history; (3) impact on daily life; (4) self-perceived psychological symptoms; and (5) unmet needs and support preferences.

The questionnaire was designed as an exploratory patient-experience instrument and did not undergo formal psychometric validation.

### Psychological assessment

Psychological outcomes reported in this study represent self-perceived symptoms rather than clinically validated psychiatric diagnoses. Participants were asked whether they had experienced symptoms such as anxiety, depressive mood, low self-esteem, or social withdrawal related to their disease experience. No validated psychological instruments (e.g., HADS, PHQ-9, GAD-7) were used.

Accordingly, findings should be interpreted as patient-reported perceptions of psychological distress rather than prevalence estimates of psychiatric disorders.

### Data collection

Participants completed the questionnaire electronically via secure online links distributed through the patient community’s official account. Informed consent was electronically obtained from all participants, ensuring confidentiality and anonymity throughout data collection.

### Statistical analysis

Descriptive statistical methods summarized demographic characteristics, clinical features, psychosocial burdens, and patient-reported needs. Categorical variables were presented as frequencies and percentages, while continuous variables were expressed as medians and interquartile ranges (IQRs) due to skewed data distributions.

Subgroup analyses included comparisons of diagnostic delays, disease impact severity, and psychosocial burdens by gender, age groups, and clinical features (e.g., patients with and without precocious puberty, those who experienced misdiagnosis). Statistical comparisons utilized Chi-square tests for categorical variables and Mann-Whitney U tests for non-parametric continuous variables. Data analysis was performed using IBM SPSS version 26.0 (IBM Corp., Armonk, NY, USA). Statistical significance was defined as a p-value < 0.05.

### Ethical approval

This study adhered to the Declaration of Helsinki guidelines. Ethical approval was granted by the Institutional Review Board (IRB) of the First Affiliated Hospital of Zhejiang University School of Medicine (Approval No.2023-0629). Electronic informed consent was obtained from all participants before survey initiation.

## Results

### Participant characteristics

A total of 152 individuals with a confirmed diagnosis of MAS participated in the survey. The cohort included 94 females (61.84%) and 58 males (38.16%), with a median age of 10.08 years (interquartile range [IQR]: 7.79–14.96 years) at the time of survey completion. Among respondents, 93.42% (142/152) were under 18 years of age.

Patients were geographically distributed across 28 of the 34 provincial-level administrative regions in China, including both urban and rural areas, indicating broad geographic coverage.

The majority of participants (72.36%) reported onset of symptoms before the age of 6 years, with a median age at initial presentation of 5.50 years (IQR: 2.96–9.46), and a median age at definitive diagnosis of 6.00 years (IQR: 3.71–10.00).

The socioeconomic profile varied widely: 44.74% of families reported monthly medical expenditures between 1,001–5,000 RMB (approximately USD 140-700), while 42.76% spent ≤1,000 RMB, indicating a significant economic burden in a substantial proportion of patients (Table 1).

### Clinical manifestations and first symptoms

MAS presented with a wide spectrum of clinical manifestations in the surveyed population, reflecting the classical triad and extended endocrine involvement. The most commonly reported initial symptom was skeletal involvement, including limb pain, deformity, or pathological fractures, affecting 61.18% (93/152) of patients. This was followed by peripheral precocious puberty, which was more prevalent among females (55/94, 58.51%) than males (1/58, 1.72%), with an overall frequency of 36.8%. Café-au-lait skin pigmentation was reported as a first symptom in 37.50% of all cases.

**Fig. 1:**
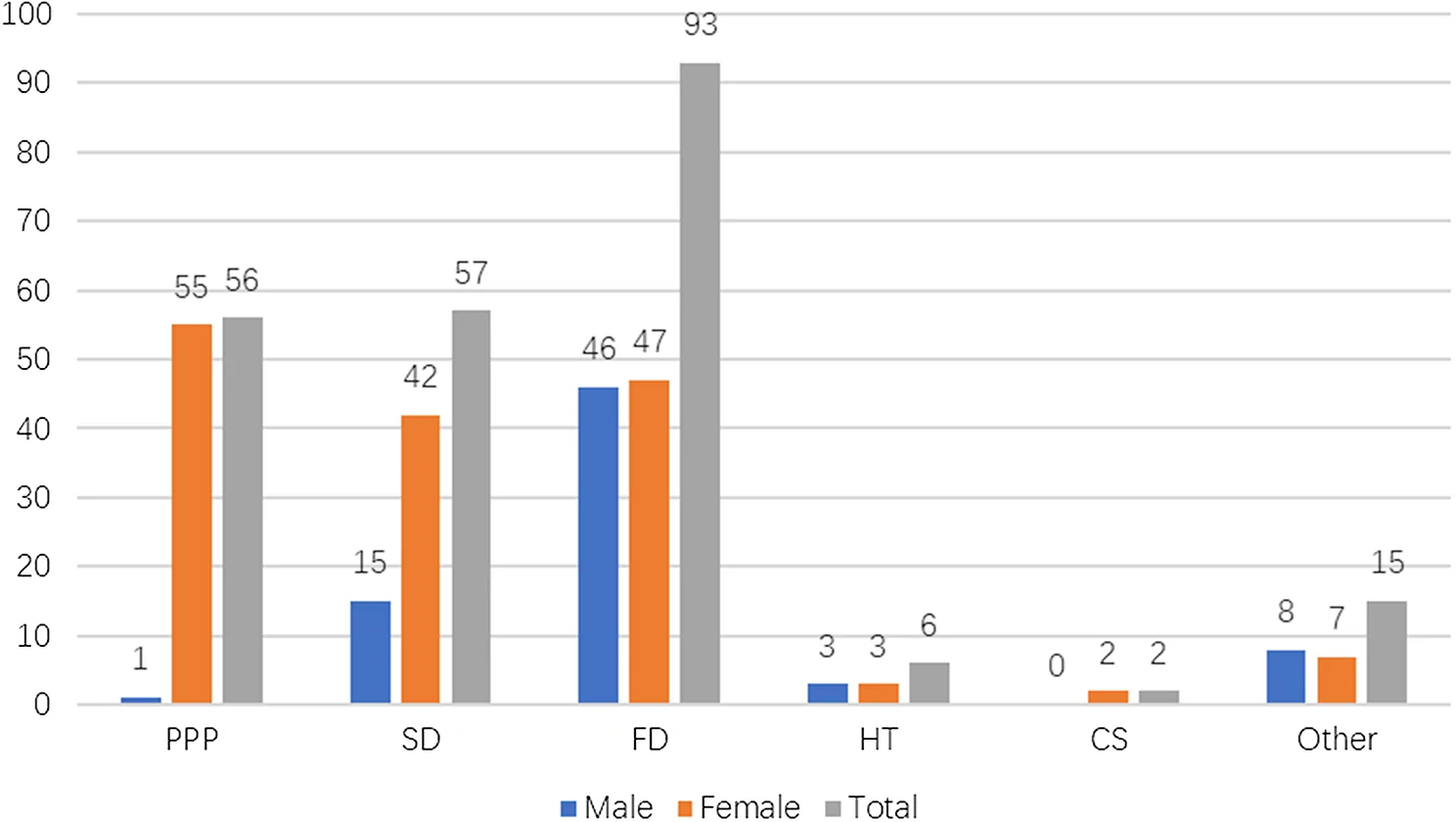
Distribution of First Symptoms among MAS Patients (n = 152)SD skin dysplasia (café au lait spots), FD fibrous dysplasia, PPP peripheral precocious puberty, HT hyperthyroidism, CS Cushing's syndrome

Less frequent but important endocrine features at onset included hyperthyroidism (3.94%) and Cushing syndrome (1.32%). In addition, 9.87% of respondents reported "other symptoms" as their initial presentation, such as headache, abnormal growth, or unspecific fatigue (Fig 1).

Bone involvement was the most common initial manifestation of MAS in this cohort. Peripheral precocious puberty showed a marked sex-specific distribution (female predominance), while café-au-lait pigmentation was also frequently reported as an initial sign.

### Diagnostic timeline and misdiagnosis

The time from initial symptom onset to confirmed diagnosis varied among participants. The median diagnostic delay was 0 months (IQR: 0–6). However, 21.05% (32/152) of respondents reported delays of ≥12 months, and some reported delays exceeding 5 years; the longest reported delay was >20 years.

**Fig. 2:**
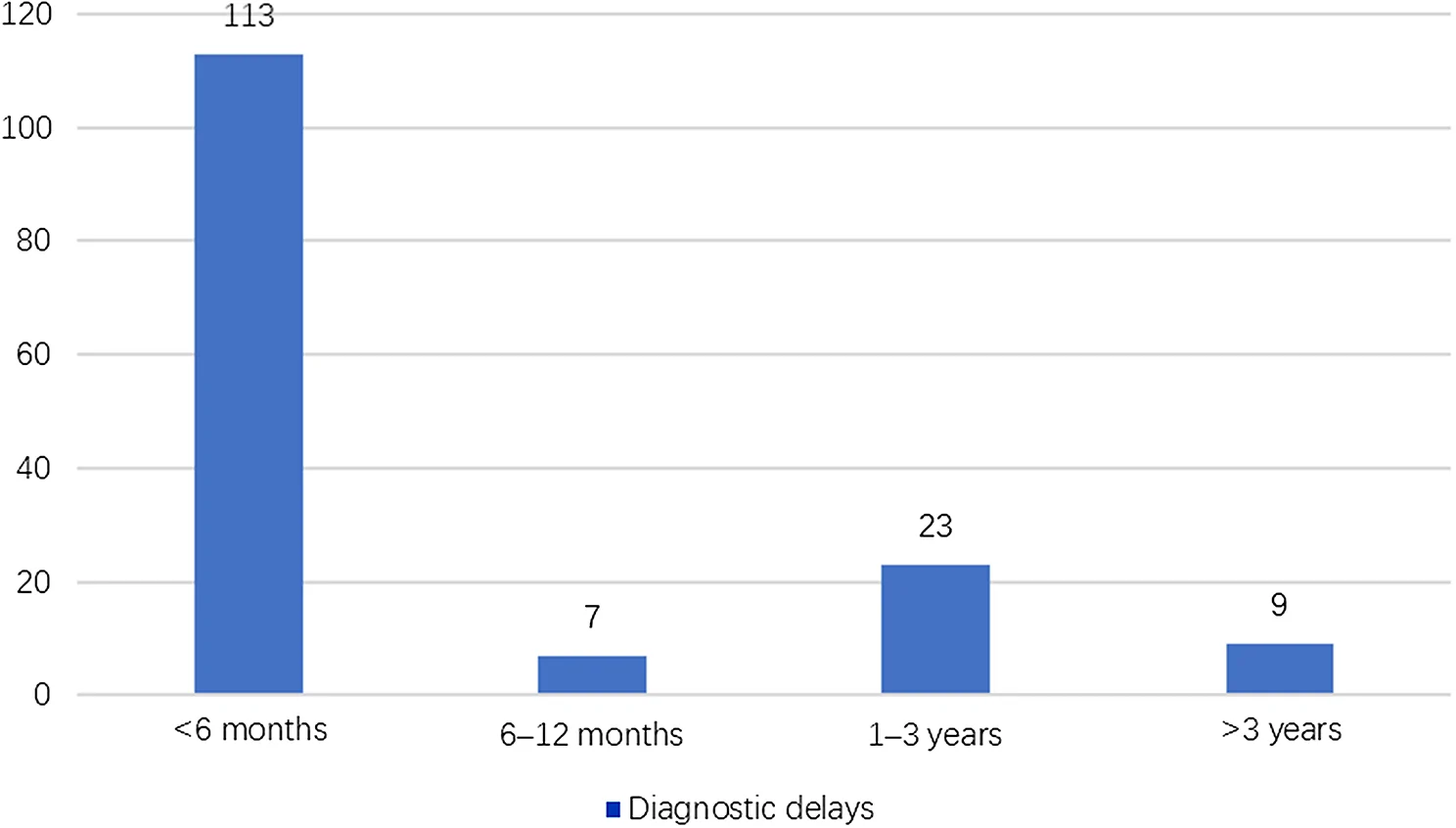
Diagnostic Delay Distribution among MAS Patients

A significant proportion of patients encountered diagnostic confusion prior to receiving a correct MAS diagnosis, with 27.63% (42/152) reporting a history of misdiagnosis. The most common preliminary misdiagnoses included isolated precocious puberty, bone tumors, vitiligo, and idiopathic scoliosis. Several patients also reported undergoing unnecessary endocrine or orthopedic interventions before MAS was ultimately recognized as a systemic disorder (Fig 2, Table 2).

### GNAS testing and molecular diagnosis

Among 152 surveyed patients, 74 (48.68%) underwent GNAS testing. Pathogenic variants were identified in 27 patients, yielding a positivity rate of 36.49% among those tested. The most frequent variants were p.R201C (n=19, 70.37%) and p.R201H (n=7, 25.92%); other reported variants included one dual p.R201H/p.R201S.

GNAS testing was not uniformly available across regions, and several participants reported cost or access barriers.

### Impact on life and psychological burden

Respondents rated MAS-related disruption in six domains of life using a five-point Likert scale (1 = no impact, 5 = extreme impact). The mean scores were highest for family life and financial burden (3.21), followed by overall quality of life (3.13) and physical mobility (2.97) (Table 3)

In terms of functional impact, 28.29% of participants rated MAS as having a severe or extreme impact (score ≥4) on their ability to study or work. Significant limitations in physical activities, such as walking or running, were reported by 38.15% of respondents. Furthermore, 46.05% experienced moderate-to-severe difficulties in social interactions or self-confidence. Notably, nearly one-fifth of patients (21.05%) perceived MAS to have an extremely negative effect on their overall quality of life.

In terms of psychological burden, 78.29% of respondents reported experiencing anxiety-related symptoms, while 37.50% reported symptoms of depression. Feelings of inferiority were noted by 36.18% of patients, and 28.95% experienced social phobia or withdrawal. Notably, only 15.79% of participants indicated that they did not experience any psychological distress (Table 4).

Despite the high prevalence of emotional difficulties, fewer than 20% of participants reported having accessed professional psychological counseling.

### Treatment patterns and unmet needs

Treatment modalities varied considerably across the MAS cohort. The most commonly reported approaches were regular follow-up observation (62.50%), anti-fibrous-dysplasia agents (38.82%), and surgical interventions (36.18%). Only a minority of patients received hormonal therapy (10.53%), typically for endocrine manifestations such as peripheral precocious puberty or hyperthyroidism (Table 5).

Monthly medical expenditures reflected a significant economic burden for MAS families: 42.76% spent ≤1,000 RMB per month on MAS-related healthcare, 44.74% reported monthly expenses between 1,001 and 5,000 RMB, and approximately 12.50% of families incurred costs exceeding 5,000 RMB per month (Table 1).

Despite this burden, patient satisfaction with treatment effectiveness remained modest. On a five-point scale, 54.61% of respondents rated the treatment effect as “neutral,” 17.11% expressed dissatisfaction, and only 28.29% reported satisfaction or higher levels of satisfaction with their current treatment (Table 6).

Despite ongoing treatment, substantial unmet needs remained among patients with MAS and their families. More than 90% of respondents (91.45%) expressed a need for greater access to specialist medical resources, making this the highest-ranked priority. Improved medical insurance reimbursement was requested by 79.61% of participants, reflecting the considerable financial burden associated with long-term disease management. In addition, 46.05% wished to participate in peer-support communities, 40.13% requested more patient education activities, and 36.84% reported a need for professional psychological counseling (Table 7).

These findings indicate that patients require not only medical treatment but also comprehensive psychosocial support, disease education, and improved access to coordinated rare disease services.

### Regional distribution

Respondents clustered in coastal provinces, led by Jiangsu (12.5%), Zhejiang (8.55%), Shandong (6.58%), Guangdong (5.92%), and Shanghai (4.61%). Several inland provinces—for example Hubei (5.26%), Sichuan (3.95%), and Henan (2.63%)—each contributed a smaller share.

## Discussion

This national cross-sectional survey provides real-world data on the diagnostic journey, disease burden, psychosocial experiences, and support needs of patients with MAS in China. By recruiting 152 patients across 28 of 34 provincial-level regions, the study offers a broad overview of patient-reported experiences in the Chinese context and helps identify potentially actionable gaps in care. Taken together, our findings point to the value of care models that integrate multidisciplinary clinical management with education, psychological support, and peer/community resources for MAS patients and families in China.

Consistent with prior international experience, our data reveal that a significant proportion of Chinese MAS patients continue to experience diagnostic delay and misdiagnosis. In our cohort, 21.05% of patients reported a diagnostic lag of more than one year, and 27.63% were misdiagnosed at least once—figures highly consistent with those observed in large European and UK registries, where diagnostic delay remains a defining feature of the so-called “diagnostic odyssey” for rare diseases [4,7]. Song et al. demonstrated a median delay of 2 years in the UK, and the EURORDIS survey found that 25% of rare disease patients in Europe wait over 5 years for an accurate diagnosis, with up to 40% initially misdiagnosed [4].

However, the specific context in China further exacerbates these challenges. The country’s vast geography, stark urban–rural healthcare disparities, and still-maturing rare disease infrastructure mean that many families initially seek care in fragmented, tiered healthcare systems with little coordination between primary, secondary, and tertiary centers. Many patients in our study described “doctor shopping” across multiple hospitals—often being evaluated for single-organ symptoms (e.g., isolated bone pain, precocious puberty, or café-au-lait macules)—without systemic recognition of MAS. This lack of a unified referral and data-sharing system, especially in resource-limited provinces, increases the risk of repeated missed diagnoses and inconsistent management, a pattern also noted in rare disease cohorts from other large, decentralized health systems.

Although molecular confirmation is increasingly used in specialized centers, current international guidelines continue to emphasize that MAS remains primarily a clinical diagnosis because of somatic mosaicism [8–11]. Less than half of our cohort accessed GNAS analysis, echoing recent reports from Asia and Latin America, where advanced molecular diagnostics are often restricted to urban or academic centers and not reimbursed by basic medical insurance.

The clinical features of MAS in this Chinese cohort align broadly with those seen in international registries, but several unique patterns are evident. While bone manifestations and peripheral precocious puberty remain the most common presentations, our data reveal a notably high rate of bone-predominant cases (61.18%), possibly reflecting a referral bias toward orthopedic rather than endocrine units. International studies report similar referral channel–driven variation, with the NIH and Korean groups noting that the proportion of patients identified through endocrinology may show higher rates of hyperfunctioning endocrinopathies [4–5].

It is also worth noting that, as in other populations, clinical heterogeneity remains the rule: nearly half of our respondents eventually developed symptoms in more than one major system. Although over 48% underwent GNAS mutation testing, the diagnostic yield was below 40%, reflecting both technical challenges (e.g., low-level mosaicism in blood, the need for affected tissue testing) and real-world limitations (non-standardized assay methods, lack of repeat testing) [12–13].

These observations highlight that a high index of clinical suspicion is required when evaluating children with recurrent fractures, early puberty, or skin pigmentation—particularly in the absence of trauma or clear family history. Second, greater standardization and reimbursement for sensitive molecular testing, such as droplet digital PCR or cfDNA analysis, are needed to reduce false negatives and missed opportunities for diagnosis [10–11].

Beyond the biomedical complexity, MAS imposes a chronic burden that is not fully captured by clinical metrics alone. Our data show that over 78% reported anxiety-related symptoms, and over one-third reported depressive symptoms, social withdrawal, or feelings of inferiority—mirroring international reports describing increased psychological burden.

Bas C. J. Majoor et al. found that negative illness perceptions, unpredictability of disease course, and persistent pain were strongly linked to diminished mental and social functioning [14]. However, in China, these issues are amplified by both cultural and systemic factors. Mental health remains stigmatized in many families, especially for children with “invisible” symptoms such as anxiety, depressive symptoms, or low self-esteem. In our cohort, fewer than 20% of patients ever accessed professional counseling—a gap driven not only by cultural reticence but also by a shortage of pediatric mental health professionals, especially outside major cities. These findings call for the integration of routine psychological screening and evidence-based interventions into MAS care pathways, a model that has shown promise in international rare disease networks but remains rare in most of China.

Interpretation of these psychological findings requires caution. First, the study did not include a control group; therefore, we cannot determine whether MAS increases the risk of anxiety or depressive symptoms compared with the general population or with other rare diseases. Second, our questionnaire captured self-perceived psychological distress (checkbox items) rather than validated diagnostic instruments, which may inflate prevalence estimates and limits direct comparability across studies. Nevertheless, population-based meta-analyses in Chinese children/adolescents using validated screening tools have generally reported lower pooled prevalence estimates for anxiety and depressive symptoms (typically around 20–30%, depending on period and instrument). In addition, studies in other rare disease settings in China indicate substantial psychological burden among family caregivers, supporting the need to integrate mental health screening and accessible support into rare disease care pathways.

A distinctive challenge for MAS patients in China is the persistent inequality in access to care and financial protection. Our survey revealed that nearly half of families spent more than 1,000 RMB per month on direct medical costs, with over 12% exceeding 5,000 RMB monthly—burdens that are especially difficult for rural and low-income households. Medical insurance coverage for MAS-related diagnostics, treatments, and follow-up is not uniform across provinces, with some families forced to pay out-of-pocket for key tests and drugs.

These findings parallel the “financial toxicity” now well-documented for rare disease families in China, who often face the double jeopardy of high direct costs and lost income due to time spent seeking care, managing disability, or providing home support. In this regard, policy initiatives such as the National Rare Disease List, regional reimbursement pilots, and disease registries are necessary but not yet sufficient—true equity will require robust national funding streams, risk-sharing for high-cost therapies, and greater cross-provincial standardization of rare disease benefits.

Encouragingly, our study highlights the growing impact of Chinese patient organizations and digital health initiatives. Respondents expressed strong preferences for educational formats such as short science videos, live expert lectures, and online peer communities—a finding consistent with other rare disease engagement studies in China and internationally.

Patient organizations and digital health platforms represent a scalable solution to China’s regional disparities, offering patient-centered education, peer support, and even telemedicine consults to families unable to travel to major hospitals. In Europe, the US, and Japan, similar organizations have been critical partners in rare disease care improvement, helping co-design registries, education programs, and research agendas [15]. For China, the next step will be formal integration of such peer-based services, and national quality standards for rare disease information.

The global rare disease community increasingly recognizes that early diagnosis, patient empowerment, and multidisciplinary, lifelong care are essential for improved outcomes [15]. The experience of European Reference Networks (ERNs) demonstrates the value of centralized expertise, cross-sector collaboration, and standardized pathways for care and research [16–18].

China’s ongoing rare disease policy reforms—including inclusion of MAS in the national rare disease centers, and pilot reimbursement programs—are promising but must now be translated into real-world improvements for families across all provinces. This will require not only technical investments in diagnostics and multidisciplinary teams, but also a cultural shift toward patient voice, holistic support, and equity in access regardless of geography or income.

## Strengths and limitations

Several limitations should be considered when interpreting our findings. First, participants were recruited through a national patient community and referral network, which may have introduced selection bias. Patients who are actively engaged with rare disease organizations may be more likely to seek specialist care, undergo genetic testing, and report unmet needs, potentially limiting generalizability to the broader MAS population. Second, this recruitment strategy may have resulted in overrepresentation of younger patients and families who were more actively engaged with patient organizations and specialist care. More than 90% of respondents were younger than 18 years of age, reflecting both the pediatric predominance of MAS and the characteristics of the recruitment platform. Consequently, adult experiences may be underrepresented. Third, psychological outcomes were based on self-reported symptoms rather than validated psychological instruments and should not be interpreted as estimates of clinical psychiatric disorders. Fourth, clinical information was not independently verified through medical record review and may be subject to recall bias and reporting inaccuracies. Finally, although the questionnaire underwent expert review and pilot testing, formal psychometric validation was not performed. Therefore, the findings should be considered exploratory and hypothesis-generating.

## Future directions

Future initiatives will include recurring academic workshops, patient–physician forums, peer-support activities, and science communication programs. We will evaluate their impact prospectively.

Future studies should incorporate longitudinal follow-up of patient-reported outcomes to better understand disease progression and long-term impacts. The use of validated psychosocial and quality of life instruments will help standardize assessments across cohorts. Additionally, research should explore genotype-phenotype correlations using high-sensitivity molecular assays, and develop intervention studies to evaluate the effectiveness of patient education, counseling, and care coordination models. Finally, cross-country comparative studies are needed to benchmark MAS care in China against international health systems and identify best practices for improving patient outcomes. Larger multicenter studies are warranted to identify predictors of diagnostic delay, misdiagnosis, and psychological burden.

## Conclusion

This national survey suggests that Chinese individuals with MAS experience substantial clinical, psychosocial, and healthcare-related challenges. While many patients achieved timely diagnosis, a significant minority continued to experience diagnostic delay, misdiagnosis, and unmet support needs. Future studies using validated patient-reported outcome measures and longitudinal follow-up are warranted to better understand the evolving needs of this rare disease population.

Addressing these gaps will not only shorten the “diagnostic odyssey” but also improve quality of life, functional outcomes, and psychosocial well-being for individuals and families living with MAS in China. As China moves toward more equitable and patient-focused rare disease management, the experiences and should help inform future clinical practice, policy, and research priorities.

**Table 1:** Demographic and Geographic Characteristics of the Surveyed MAS Patients

Age(year)	Male (58/38.16%)	Female (94/61.84%)	Total (152/100%)
	9.63[7.92,13.50]	10.46[7.75,18.17]	10.08[7.79,14.96]
Age at first symptoms(year)	5.96[3.33,10.00]	5.00[2.67,8.00]	5.50[2.96,9.46]
Age at final diagnosis(year)	5.96[3.92,10.00]	6.00[3.50,9.17]	6.00[3.71,10.00]
Time to diagnosis	0.00[0.00,0.00]	0.00[0.00,1.00]	0.00[0.00,0.50]
Monthly treatment cost (yuan)			
≤ 1000	28(48.28%)	37(39.36%)	65(42.76%)
1001–5000	24(41.38%)	44(46.81%)	68(44.74%)
5001–10,000	4(6.90%)	11(11.70%)	15(9.87%)
≥ 10,001	2(3.45%)	2(2.13%)	4(2.63%)
Misdiagnosis			
Yes	15(25.86%)	27(28.72%)	42(27.63%)
No	43(74.14%)	67(71.28%)	110(72.37%)

**Table 2:** Diagnostic Journey

Variable	*n* (%)
Delay 0–6 months	113(74.34%)
Delay 6–12 months	7(4.61%)
Delay 1–3 years	23(15.13%)
Delay > 3 years	9(5.92%)
Misdiagnosis	42 (27.63%)

**Table 3:** Perceived Impact of MAS on Daily Life Domains

Ability to study or work	No impact	Little impact	Moderate impact	Severe Impact	Extreme impact	Average score
	31(20.39%)	38(25%)	40(26.32%)	16(10.53%)	27(17.76%)	2.8
Physical activities	35(23.03%)	33(21.71%)	26(17.11%)	17(11.18%)	41(26.97%)	2.97
Social interactions /self-confidence	40(26.32%)	42(27.63%)	30(19.74%)	18(11.84%)	22(14.47%)	2.61
Emotional stability	32(21.05%)	42(27.63%)	37(24.34%)	19(12.5%)	22(14.47%)	2.72
Family life and financial burden	17(11.18%)	34(22.37%)	35(23.03%)	32(21.05%)	34(22.37%)	3.21
Overall quality of life	15(9.87%)	40(26.32%)	39(25.66%)	26(17.11%)	32(21.05%)	3.13

Data are presented as n (%). Impact was rated on a 5-point Likert scale (1 = no impact, 2 = little, 3 = moderate, 4 = severe, 5 = extreme). The average score was calculated as Σ(level × frequency)/N; higher scores indicate greater perceived impact. Valid respondents: *N* = 152. Percentages may not sum to 100% due to rounding. Abbreviations: MAS, McCune–Albright syndrome; QoL, quality of life

**Table 4:** Self-perceived Psychological Symptoms

Psychological Symptom	Number of Patients (%)
Anxiety-related symptoms	119 (78.29%)
Depressive symptoms	57 (37.50%)
Low self-esteem	55 (36.18%)
Social withdrawal/phobia	44 (28.95%)
No psychological distress	24 (15.79%)
Other	9 (5.92%)

**Table 5:** Distribution of Current Treatment Modalities in MAS Patients

Treatment Modality	Number of Patients (%)
Regular follow-up/observation	95 (62.50%)
Anti-fibrous dysplasia medications (primarily bisphosphonates)	59 (38.82%)
Surgery	55 (36.18%)
Hormonal therapy	16 (10.53%)
Other	22 (14.47%)

**Table 6:** Patient Satisfaction with Treatment Effectiveness among MAS Patients (*n* = 152)

Satisfaction Level	Number of Patients (%)
Very dissatisfied	10 (6.58%)
Dissatisfied	16 (10.53%)
Neutral	83 (54.61%)
Satisfied	35 (23.03%)
Very satisfied	8 (5.26%)

**Table 7:** Patient-Reported Priority Support Needs

Support Need	Number of Patients (%)
More professional medical resources	139 (91.45%)
Medical insurance reimbursement policy	121 (79.61%)
Patient education (online/offline)	61 (40.13%)
Professional psychological counseling	56 (36.84%)
Peer-support communities	70 (46.05%)
Other	8 (5.26%)

## Supplementary Information

Below is the link to the electronic supplementary material.

Supplementary Material 1

## Data Availability

De-identified participant data, a data dictionary, and analysis code are available from the corresponding author (Chunlin Wang, MD/PhD; hzwangcl@zju.edu.cn.) upon reasonable request during peer review and will be deposited in an open repository (e.g., OSF/Zenodo) upon acceptance.
