# Supplementary material for: Diagnostic delay, misdiagnosis, and patient-reported psychosocial burden among Chinese individuals with McCune–Albright syndrome: a national cross-sectional survey
Source: Orphanet J Rare Dis. 2026 Jul 15;21:355. doi: 10.1186/s13023-026-04507-y (PMC13656380; doi:10.1186/s13023-026-04507-y)
Supplement: Supplementary file 1 [file 13023_2026_4507_MOESM1_ESM.pdf]

Supplementary Appendix：

Structured Questionnaire Used in the National Survey of McCune–Albright Syndrome (MAS) Patients in China

Welcome!

Thank you very much for taking the time to participate in this questionnaire.

This survey is jointly conducted by the McCune–Albright Syndrome Clinical Research Consortium (MASCRC) and the Bone Care Center, a national patient-support organization. Together, we are committed to advancing clinical research on McCune–Albright syndrome (MAS), improving multidisciplinary care, and enhancing the quality of life of individuals living with MAS.

The purpose of this survey is to better understand the real-world experiences of patients with MAS, including their diagnostic journey, treatment experiences, daily challenges, quality of life, and unmet healthcare needs. Your participation will help us optimize clinical care pathways, improve patient-centered services, and promote future research and healthcare policy for this rare disease.

All information you provide will be kept strictly confidential and used exclusively for research purposes. No personally identifiable information will be disclosed in any publication or presentation.

Completion of this questionnaire will take approximately 10–20 minutes. Every response is valuable and will contribute to improving the diagnosis, treatment, and long-term support of individuals living with MAS.

If you agree to participate in this study, please read the informed consent information carefully and indicate your consent before clicking "Start" to begin the questionnaire.

Thank you again for your generous support and participation.

Domain 1. Demographic and Socioeconomic Characteristics

1. Participant name (optional)

____

2. Age (years)

____

3. Date of birth (YYYY/MM/DD)

____

4. Place of birth

____

5. Telephone number (optional)

6. Gestational age at birth (weeks)

7. Birth order

8. Parity

9. Birth weight (kg)

□ Unknown

10. Current height (cm)

11. Current weight (kg)

12. Sex

□ Male
□ Female

13. Maternal infection during pregnancy

□ Yes
□ No
□ Unknown

14. Maternal radiation exposure during pregnancy

□ Yes
□ No
□ Unknown

15. Birth asphyxia

□ Yes
□ No
□ Unknown

16. Father's height (cm)

17. Father's weight (kg)

18. Father's health status

19. Mother's height (cm)

20. Mother's weight (kg)

21. Mother's health status

Domain 2. Clinical Manifestations and Diagnostic History

22. Age at first medical consultation (years/months)

23. Age at confirmed diagnosis (years/months)

24. Hospital or institution where MAS was diagnosed

25. Height at diagnosis (cm)

26. Weight at diagnosis (kg)

27. Bone age at diagnosis

□ Known (please specify)
□ Unknown

28. Evidence of pubertal development at diagnosis

□ Yes (please specify)
□ No

□ Unknown

29. First manifestation(s) of MAS (select all that apply)

□ Peripheral precocious puberty
□ Café-au-lait macules
□ Fibrous dysplasia-related skeletal manifestations
□ Hyperthyroidism
□ Cushing syndrome
□ Other (specify)

30. Age at onset of first manifestation

____

31. Other manifestations

____

32. Age at onset of additional manifestations

____

33. Abnormal imaging findings (select all that apply)

□ Ovarian ultrasonography
□ Thyroid ultrasonography
□ Testicular ultrasonography
□ Other imaging findings

34. Anatomical sites affected by fibrous dysplasia

____

35. Methods used to evaluate fibrous dysplasia

____

36. Have you undergone GNAS genetic testing?

□ Yes
□ No

37. Other genetic testing performed

____

38. Do you believe you experienced a misdiagnosis before receiving a confirmed diagnosis of MAS?

□ Yes
□ No

Domain 3. Impact on Daily Life

39. Please rate the impact of MAS on each domain (1 = No impact; 5 = Extreme impact).

Domain	1	2	3	4	5
Ability to study/work	○	○	○	○	○
Physical activity (walking, standing, running)	○	○	○	○	○
Social interaction/self-confidence	○	○	○	○	○
Emotional stability	○	○	○	○	○
Family life and financial burden	○	○	○	○	○
Overall quality of life	○	○	○	○	○

What aspect of daily life has been most affected by MAS?

____

Domain 4. Treatment and Self-perceived Psychological Symptoms

40. Current treatment modalities (select all that apply)

□ Hormonal therapy
□ Anti-fibrous dysplasia medications (primarily bisphosphonates)
□ Surgery
□ Regular follow-up/observation
□ Other (specify)

41. Average monthly medical expenditure

□ ≤1,000 RMB
□ 1,001–5,000 RMB
□ 5,001–10,000 RMB
□ ≥10,001 RMB

42. Main treatment-related difficulties (select all that apply)

□ High treatment costs
□ Limited access to specialist physicians
□ Treatment-related adverse effects
□ Limited healthcare facilities
□ Other (specify)

43. Please rate your satisfaction (1 = Very dissatisfied; 5 = Very satisfied).

Item	1	2	3	4	5
Treatment effectiveness	○	○	○	○	○
Medical service attitude	○	○	○	○	○
Accessibility of specialized medical care	○	○	○	○	○

44. Have you experienced any of the following? (select all that apply)

□ Anxiety-related symptoms
□ Depressive symptoms
□ Low self-esteem
□ Social withdrawal/social anxiety
□ No psychological distress
□ Other (specify)

Domain 5. Unmet Needs and Support Preferences

45. Which types of support do you currently consider most important? (select all that apply)

□ Improved medical insurance reimbursement
□ Regular patient education activities
□ Professional psychological counseling
□ Peer-support communities
□ Greater access to specialist medical resources
□ Other (specify)

46. Which patient-support activities would you be most willing to participate in?

□ Educational lectures
□ Patient-support groups
□ Professional psychological counseling
□ Online consultation
□ Other (specify)

47. Preferred formats for patient education (select all that apply)

□ Face-to-face educational forums
□ Live-streaming educational sessions
□ Short educational videos
□ Written educational materials
□ Other (specify)

48. Please provide any suggestions for improving MAS diagnosis, treatment, or patient services.

____

Translation Statement

The questionnaire was originally developed in Chinese. The present English version was translated by the study investigators for publication purposes. It is provided to facilitate interpretation of the study and has not undergone independent linguistic validation.
